# Dr. Robert G. Wilding

**Published:** 1904-03

**Authors:** 


Dr. Robert G. Wilding, of Malone, N. Y., a permanent member of the Medical Society of the State of New York, died suddenly, February 24, 1904, of an affection of the heart, aged 67 years. Dr. Wilding was a man of prominence both in professional life and as a citizen, and will be greatly missed in both spheres of great usefulness.

